# RBM10 Deficiency Is Associated With Increased Immune Activity in Lung Adenocarcinoma

**DOI:** 10.3389/fonc.2021.677826

**Published:** 2021-07-21

**Authors:** Bing Liu, Yaqi Wang, Han Wang, Zhongwu Li, Lujing Yang, Shi Yan, Xin Yang, Yuanyuan Ma, Xuan Gao, Yanfang Guan, Xin Yi, Xuefeng Xia, Jingjing Li, Nan Wu

**Affiliations:** ^1^ Key Laboratory of Carcinogenesis and Translational Research (Ministry of Education), Department of Thoracic Surgery II, Peking University Cancer Hospital & Institute, Beijing, China; ^2^ Geneplus-Beijing Institute, Geneplus-Beijing, Beijing, China; ^3^ Key Laboratory of Carcinogenesis and Translational Research (Ministry of Education), Department of Pathology, Peking University Cancer Hospital & Institute, Beijing, China; ^4^ The Precision Medicine Centre of Drum Tower Hospital, Medical School of Nanjing University, Nanjing, China

**Keywords:** RBM10, deficiency, immune activity, lung adenocarcinoma, immunotherapy

## Abstract

**Introduction:**

RBM10 is one of the frequently mutated genes in lung adenocarcinoma (LUAD). Previous studies have confirmed that RBM10 could suppress the disease progression and cell proliferation in LUAD, but its loss-of-function mutations are more frequent in early-stage disease and decrease with the advancement of the clinical stage. This is contradictory to its role of tumor suppressor. Here, we conducted a systematic analysis to elucidate whether there was other potential biological significance of RBM10 deficiency during the progression of LUAD.

**Materials and Methods:**

The whole exome sequencing data of 39 tumor samples from early-stage LUADs (GGN cohort) and genomic and transcriptome data of the Cancer Genome Atlas (TCGA) LUAD cohort (TCGA_LUAD cohort) and a Chinese LUAD cohort (CHOICE_ADC cohort) were first obtained. Systematic bioinformatic analyses were then conducted to determine gene expression signature, immune infiltration levels and predicted immunotherapy response. Immunohistochemistry (IHC) was also conducted to validate the result of immune infiltration.

**Results:**

The mutation rate of RBM10 was significantly higher in the GGN cohort than that in the TCGA_LUAD and CHOICE_ADC cohorts. In both TCGA_LUAD and CHOICE_ADC cohorts, multiple immune related pathways were markedly enriched in RBM10 deficient group. Further analyses showed that tumors with RBM10 mutations displayed higher TMB, and LUADs with RBM10 deficiency also showed higher HLA expression levels, including many HLA class I and II molecules. Additionally, many immune cells, including myeloid dendritic cells, macrophages, neutrophils and CD8+T cells, showed higher infiltration levels in LUADs with RBM10 deficiency. Finally, some immune checkpoint molecules, such as PD-L1 and TIM-3, were highly expressed in RBM10 deficient population and the predicted immunotherapy response was calculated through TIDE algorithm, showing that IFNG expression, MSI score and CD8 expression were higher in RBM10 deficient group, while MDSC and M2 macrophage were lower in RBM10 deficient group.

**Conclusion:**

Our study demonstrates that RBM10 deficient LUADs show higher HLA expression and immune cell infiltration, and some immune checkpoint molecules are also highly expressed. In brief, RBM10 deficiency could enhance anti-tumor immunity in LUAD.

## Introduction

Lung cancer is the leading cause of cancer-related deaths worldwide, among which lung adenocarcinoma (LUAD) is the most common histological subtype (1). Despite advances in treatment approaches, including chemotherapy and targeted therapy, the prognosis of LUAD remains dismal, with an overall 5-year survival rate of less than 20% in the United States (1, 2). Recently, immunotherapy has significantly altered the current landscape of cancer treatment, including lung cancer (3–5). However, only a fraction of patients will benefit from immunotherapy in unselected population (6, 7). Therefore, it is imperative to unravel the molecular mechanisms associated with the development and progression of LUAD, which would improve clinical management and therapeutic strategies.

Based on current knowledge, various molecular events take place during the progression of LUAD from adenocarcinoma *in situ* (AIS) and minimally invasive adenocarcinoma (MIA), which usually present as ground-glass nodules (GGNs) on chest computed tomography (CT) scans (8, 9), to invasive adenocarcinoma (IAC). Mutational frequency of significant driver genes, such as EGFR and KRAS, increases along with the advance of clinical stage (10). Except for EGFR and KRAS, RBM10 is also one of the driver genes of LUAD (11, 12). In contrast to EGFR and KRAS, the mutations of RBM10are more frequent in early-stage LUAD and their incidence decreases with the advancement of the clinical stage, which might be an important molecular event during the development of LUAD (10, 13).

RBM10 is an RNA-binding protein and a splicing regulator located on the X chromosome, which is involved in pre-mRNA (messenger RNA) splicing and post-transcriptional regulation (14). Previous studies have confirmed that RBM10 could suppress disease progression and cell proliferation in LUAD *via* regulating the alternative splicing of NUMB and EIF4H (15, 16). Moreover, Zhao et al. verified that loss-of-function mutations in RBM10 contributed to the pathogenesis of LUAD (17). These studies suggested that LUAD with RBM10 mutations is more deleterious, which is contradictory to the observation that patients with advanced-stage LUAD harbor fewer mutations than patients with early-stage LUAD.

To uncover the ramifications of RBM10 deficiency, we performed whole exome sequencing (WES) in 39 tumor samples from early-stage LUADs presenting as GGNs. Besides, genomic and transcriptome data of the Cancer Genome Atlas (TCGA) LUAD cohort and a Chinese LUAD cohort were obtained from public databases for systematic analysis. Through systematic bioinformatic analyses and IHC validation, we found that RBM10 deficient LUADs showed higher HLA expression and immune cell infiltration, and some immune checkpoint molecules were also highly expressed. In brief, we demonstrate that RBM10 deficiency could enhance anti-tumor immunity in LUAD.

## Materials and Methods

### Patients and Samples

Thirty-seven treatment-naïve patients with early-stage LUAD who underwent radical resection *via* lobectomy or sublobectomy between December 2012 and November 2017 at the Department of Thoracic Surgery II, Peking University Cancer Hospital & Institute were recruited for WES in this study. Two of these patients had synchronous dual primary LUADs, so there were totally 39 primary tumors involved in WES. These tumors all presented as pure or mixed GGN on CT scans and were confirmed to be pathological stage I disease after surgery according to the eighth edition of the American Joint Committee for Cancer Staging System for lung cancer (18). These patients were referred to as the GGN cohort. Additionally, all tumor samples contained at least 20% of tumor cells and without adjacent normal tissue, as confirmed by pathologists. Clinicopathological characteristics of these patients are listed in **Table S1**. This study was conducted in accordance with the Declaration of Helsinki and was also approved by the Ethics Committee of Peking University Cancer Hospital & Institute (Institutional Review Board No. 2019KT59). All patients provided written informed consent before surgery.

Primary tumor and paired adjacent normal tissues were collected immediately after resection, then snap-frozen and stored at -80°C until they were used for WES. Normal tissues were usually collected at the edge of the resected lobe and macroscopically normal. In the cases of sublobar resection, they were collected > 2cm apart from the tumor edge.

### Whole Exome Sequencing (WES)

Genomic DNA was extracted from the tumor and adjacent normal tissues using the QIAamp DNA Mini Kit (cat. No. 51304, Qiagen, Hilden, Germany). Purified genomic DNA (100 ng~1 µg) from each sample was sonicated using Covaris S220. Libraries were constructed from each sample using the Agilent SureSelectXT2 kit (Illumina) according to the manufacturer’s instructions and were further captured using the Agilent SureSelect Target Enrichment System (Human All Exon V6 Kit). Paired-end sequencing of 2×150 bp fragments was performed on the Illumina HiSeq X Ten platform. The sequencing depths of the individual samples are listed in **Table S2**.

### Somatic Variant Calling

To identify somatic variations, adjacent normal tissues were used as normal controls. Somatic single-nucleotide variants (SNVs) were identified using MuTect2. The passed variants were further filtered using the following criteria to obtain a more confident set of SNVs: (1) at least five high-quality reads covering the mutation (Phred score >=30, mapping quality >=30, without paired-end reads bias); (2) the mutation was not present in >1% of the population from the 1000 Genome Project or dbSNP databases; (3) the mutation was not present in the normal samples. Somatic mutations were annotated by Ensembl Variant Effect Predictor (VEP). The SNVs and indels of each sample are listed in **Table S3**.

### Calculating Tumor Mutational Burden (TMB) and Clustering of Neoantigens

TMB is defined as the total number of nonsynonymous mutations of coding regions of a tumor genome per mega-base (Mb) of a tumor genome (19). Mutations from the 39 tumor samples were clustered into subclones using PyClone-0.13.0 based on SNVs (20), and copy number variations were analyzed using FACETS. Neoantigens were predicted by NetMHCpan-4.0 (21), and assigned to the subclones based on the mutations after which the total number of neoantigens was calculated for comparison.

### Database

For the LUAD cohort from TCGA, we filtered out the patients whose clinical information and mutation data were available. A total of 562 patients were included in this study (termed as TCGA_LUAD cohort), which is comprised of 277 stage I and 285 stage II-IV cases. The profiles of original data were downloaded at cBioPortal (https://www.cbioportal.org). As for the Chinese cohort, genomic and RNA-sequencing (RNA-seq) data of 128 patients were obtained from the study conducted by Wu et al. (termed as CHOICE_ADC cohort) (22), and this cohort is comprised of 68 stage I and 60 stage II-IV cases.

### Gene Set Enrichment Analysis

We performed Gene Set Enrichment Analysis (GSEA) to correlate the TCGA and CHOICE_ADC cohorts grouped by RBM10 expression levels to the known hallmark gene expression signatures according to the MdigDB database (http://software.broadinstitute.org/gsea/downloads.jsp), respectively. Normalized gene expression was taken as the input. We followed the GSEA user’s guide (http://www.broadinstitute.org/gsea/doc/GSEAUserGuideFrame.html) using default parameters to run the software. The FDR corrected q value < 0.05 is considered statistically significant. The single sample GSEA (ssGSEA) was analyzed using the GSVA program for immune related signatures.

### Immune Infiltration Level and Immune Therapy Response Prediction

Immune infiltration levels for each patient were estimated by TIMER with normalized gene expression data (http://timer.cistrome.org) (23). The predicted immunotherapy response was calculated through TIDE algorithm (http://tide.dfci.harvard.edu) (24).

### Immunohistochemistry

Serial 4μm sections of the formalin-fixed paraffin-embedded (FFPE) samples of the 39 tumors involved in WES (GGN cohort) were cut onto glass slides and subjected to immunohistochemistry (IHC) staining. Slides stained for CD3, CD4 and CD8 were respectively labeled by a mouse anti-CD3 monoclonal antibody (clone B1.1; ZSGB-BIO, Beijing, China), a mouse anti-CD4 monoclonal antibody (clone B486A1; ZSGB-BIO, Beijing, China) and a rabbit anti-CD8 monoclonal antibody (clone SP16; ZSGB-BIO, Beijing, China) in a working solution and incubated at 37°C on an autostainer (BenchMark ULTRA, Roche, Ventana Medical Systems, Oro Valley, AZ, USA). For each slide, manual regional annotation and machine cell counts were used to measure cell density in intra-tumoral areas. Positive cell density was analyzed by whole slide digital scanning using a Digital Pathology Scanner (Aperio VERSA, Leica Biosystems, Buffalo Grove, IL, USA), and the scoring was assessed on an Aperio Scanscope (Aperio Technologies, USA) by the method of rare event tissue test. Positive cell counts were measured in 8 intra-tumoral non-overlapping fields using fixed areas of 0.078 square millimeters.

### Statistics

All data analyses were conducted in R version 3.6.1 package and GraphPad Prism 8 (GraphPad Software, Inc). For comparison of continuous variables, the student t-test or Mann-Whitney U-test was used. For comparison of categorical variables, the χ^2^ test or Fisher’s exact test was used. The overall survival was estimated by Kaplan-Meier analysis and compared using log-rank test. Two-sided *p* value < 0.05 was considered statistically significant.

## Results

### RBM10 Presents a Higher Mutation Rate in Early-Stage LUAD and Its Mutations Lead to Functional Deficiency

**Figure 1:**
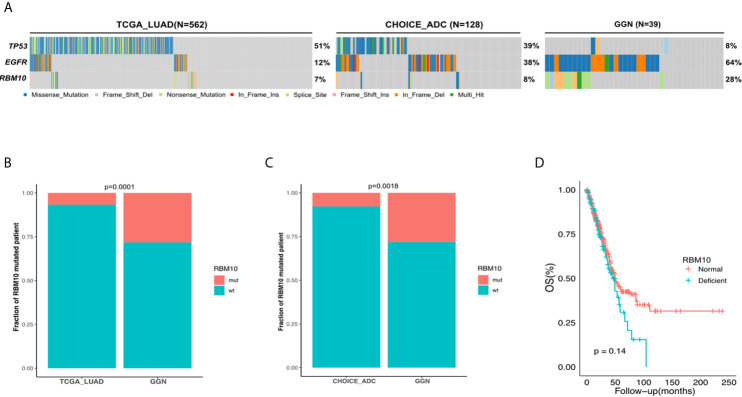
RBM10 is highly mutated in early-stage LUAD. **(A)** The mutational frequency of RBM10 in the TCGA_LUAD, CHOICE_ADC and GGN cohorts. **(B, C)** RBM10 mutation rate is significantly higher in GGN cohort than those in the TCGA_LUAD **(B)** and CHOICE_ADC **(C)** cohorts. **(D)** RBM10 deficiency leads to shorter overall survival in LUAD.

To characterize the mutation rate of RBM10 in the early and late stages of LUAD, we compared genomic data from three cohorts: the GGN cohort, the TCGA_LUAD cohort and the Chinese LUAD cohort (CHOICE_ADC cohort). The GGN cohort was composed of 39 tumor samples from 37 patients who presented with pure or mixed GGNs on CT scans and were all pathological I disease, which represented the early stage in the development of LUAD. Similar to the TCGA_LUAD cohort, the CHOICE_ADC cohort also comprised numerous locally advanced and late-stage diseases, and approximately 50% cases of this cohort were stage II-IV LUAD patients. The patient demographics of the TCGA_LUAD and CHOICE_ADC cohorts can be found in **Table S4**. The mutational frequency of RBM10 was 8% (10/128) in the CHOICE_ADC cohort, which was approximately equivalent to that in the TCGA_LUAD cohort (7%, 38/562), suggesting that RBM10 mutations wasn’t affected by racial background. However, RBM10 mutations were identified in 28% (11/39) of samples in the GGN cohort (**Figure 1A**), which was significantly higher than that in the TCGA_LUAD and CHOICE_ADC cohorts (both *P*<0.01, **Figures 1B, C**). These results indicate that RBM10 is more frequently mutated in early-stage LUAD, and its mutations are early molecular events in the pathogenesis of LUAD.

We next explored the consequence of RBM10 mutations. According to the mutational spectrum in the GGN cohort, RBM10 mutations were mainly protein-truncating variants, including nonsense and frameshift mutations, which often cause functional deficiency (**Figure S1A**). In addition, the missense mutation detected in the GGN cohort was predicted to be deleterious by SIFT (25). To further examine how RBM10 mutations affected gene expression, we compared RBM10 mRNA levels of samples harboring RBM10 mutations with those lacking RBM10 mutations in the TCGA_LUAD cohort. RBM10 mutated patients exhibited significant reduction in mRNA expression compared to wild-type patients. (*P*<0.01, **Figure S1B**). This indicates that RBM10 mutations lead to reduction in mRNA levels. To study how RBM10 deficiency would affect the tumor development, samples in the lower quartile of RBM10 mRNA expression were assigned to the deficient group, while the rest were assigned to the normal functioning group, respectively. We then explored the correlation between RBM10 expression and overall survival of LUAD patients in the TCGA_LUAD cohort and found that LUAD patients with RBM10 deficiency showed shorter overall survival, although it did not reach statistical significance (*P*=0.14, **Figure 1D**).

These results suggest that RBM10 mutations attenuate its tumor suppressive function, which is more malicious for patients. However, the frequency of RBM10 mutations is lower in patients with late-stage disease.

### Immune Related Pathways Are Enriched in LUADs With RBM10 Deficiency

**Figure 2:**
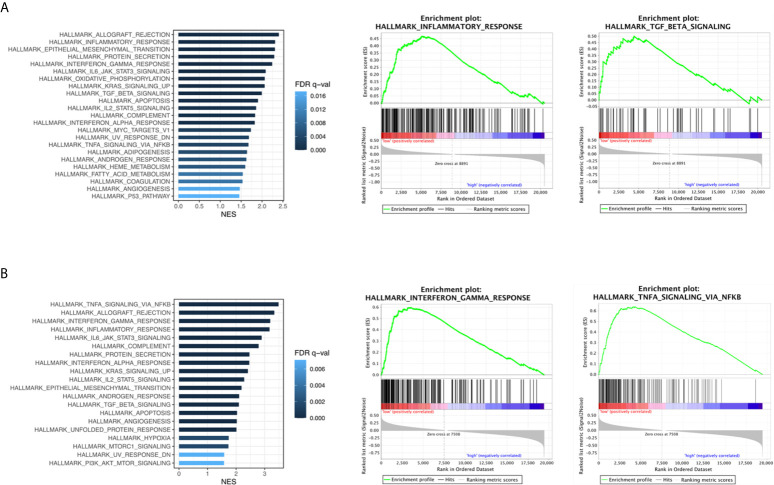
Immune related pathways enriched in RBM10 deficient LUADs. **(A)** Enriched functional pathways in RBM10 deficient LUADs in the TCGA_LUAD cohort. **(B)** Pathways enriched in RBM10 deficient LUADs in the CHOICE_ADC cohort.

To ascertain the biologically relevant changes between the RBM10 deficient and normal functioning groups, we searched for pathways enriched in both TCGA_LUAD and CHOICE_ADC cohorts (**Tables S5, S6**). Multiple immune related pathways were markedly enriched in the RBM10 deficient group in both cohorts, including the interferon-γ and interferon-α response pathways which have antitumor effect and TGF-β signaling pathway that could inhibit cell proliferation (**Figures 2A, B**). In addition, pathways including IL6/JAK/STAT3 signaling pathway and inflammatory response pathway which could promote tumorigenesis were also enriched in RBM10 deficient patients. Despite the divergent effects on tumor progression, these pathways are all related to immune response, and the results demonstrate that RBM10 deficiency might alter immunogenicity in patients with LUAD.

### RBM10 Mutations Are Associated With Elevated TMB and LUADs With RBM10 Deficiency Show Higher HLA and Cytokine Expression Levels

To further investigate how immune activity was altered in RBM10 deficient patients, we first compared TMB, which is reported to correlate with anti-tumor immunity (26). We found that tumors with RBM10 mutations displayed higher TMB levels than wild-type patients in the CHOICE_ADC cohorts (*P*=0.024, **Figure S2B**). The TMB level showed similar trend in the TCGA_LUAD cohort, although the result wasn’t statistically significant (*P*=0.098, **Figure S2A**). In the merged dataset of these two cohorts, TMB was significantly higher in the RBM10 mutant group (*P*=0.013, **Figure S2C**). These results suggest that LUAD patients with RBM10 mutations have higher TMB. As mutations in genome could drive neoantigen formation, which induced immune responses against tumor cells (27), we next calculated the predicted neoantigen in the GGN cohort through WES data. However, there was no statistically significant difference between the groups, possibly due to the small sample size (**Figures S2D–2F**).

**Figure 3:**
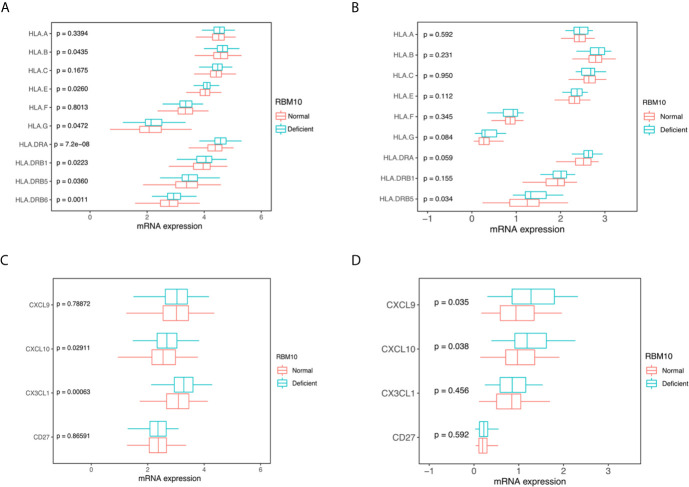
RBM10 deficiency is associated with higher HLA and cytokine expression. **(A, B)** RBM10 deficient LUADs show higher HLA expression levels in both TCGA_LUAD cohort **(A)** and CHOICE_ADC cohort **(B)**. **(C, D)** RBM10 deficiency is also associated with elevated cytokines in both TCGA_LUAD **(C)** and CHOICE_ADC cohorts **(D)**.

Human leucocyte antigens (HLA) play a crucial role in anti-tumor immunity, which can drive antigen presentation (28). There are three classes of HLA: class I, II and III. Among them, HLA I and II are the two major types of the HLA complex, which accommodates peptide antigens presented by antigen-presenting cells. We compared HLA expression between LUAD patients with RBM10 deficiency and those with normal expression. In the TCGA_LUAD cohort, the expression of HLA-G, HLA-E and HLA-B, all of which belong to HLA class I molecules, were significantly higher in LUADs with RBM10 deficiency (all *P*<0.05, **Figure 3A**). Certain HLA class II molecules, including HLA-DRB6, HLA-DRB5, HLA-DRB1 and HLA-DRA, had a higher expression in RBM10 deficient samples (all *P*<0.05, **Figure 3A**). Similarly, we also found that HLA-G, HLA-DRA and HLA-DRB5 were highly expressed in RBM10 deficient group in the CHOICE_ADC cohort, although only the result of HLA-DRB5 reached statistical significance (*P*=0.034, **Figure 3B**). Additionally, expression of several cytokines was also higher in RBM10 deficient samples, including CXCL10 and CX3CL1 in the TCGA cohort and CXCL9 and CXCL10 in the CHOICE_ADC cohort, respectively (all *P*<0.05, **Figures 3C, D**). The higher levels of expression of HLA molecules and cytokines indicate that the antigen presentation and immune activity are more active in LUADs with RBM10 deficiency.

### Immune Infiltration Levels Are Higher in LUADs With RBM10 Deficiency

**Figure 4:**
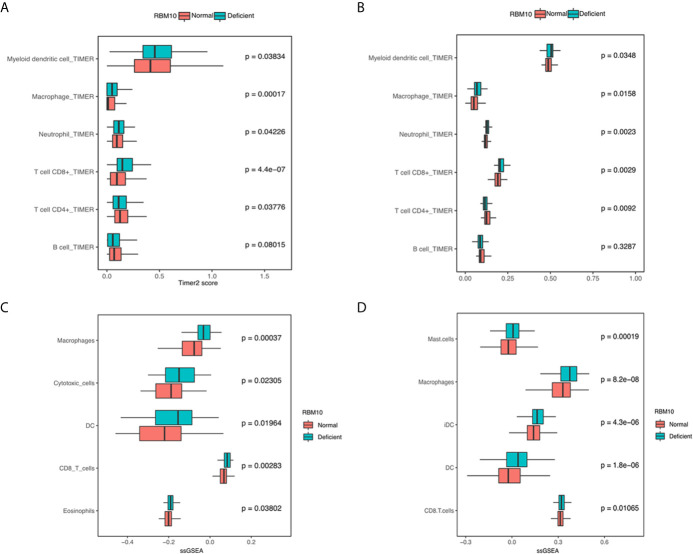
LUADs with RBM10 deficiency show higher immune infiltration levels. **(A, B)** Immune infiltration levels estimated by TIMER are higher in RBM10 deficient LUADs in both TCGA_LUAD **(A)** and CHOICE_ADC **(B)** cohorts. **(C, D)** Diverse immune signatures estimated by ssGSEA also show higher enrichment levels in RBM10 deficient LUADs in TCGA_LUAD **(C)** and CHOICE_ADC **(D)** cohorts.

Apart from antigen presentation, anti-tumor immunity relies on the activity of immune cells as well. Thus, we evaluated whether RBM10 expression correlated with the extent of immune infiltration in LUAD. We found that myeloid dendritic cells, macrophages, neutrophil and CD8+T cells consistently showed significantly higher infiltration levels in LUADs with RBM10 deficiency in both TCGA_LUAD and CHOICE_ADC cohorts (all *P*<0.05, **Figures 4A, B**). Besides, LUADs with RBM10 deficiency exhibited lower CD4+T cell and B cell infiltration in both cohorts. Similar results were found by ssGSEA, where diverse immune signatures, including those associated with macrophages, cytotoxic cells, DC cells and CD8+T cells, showed significantly higher enrichment levels in RBM10 deficient LUADs in both TCGA_LUAD and CHOICE_ADC cohorts (all *P*<0.05, **Figures 4C, D**). These results indicate that RBM10 deficiency may affect immune cell recruitment in LUAD. Moreover, CD8+ T cells, which were mainly associated with anti-tumor activity, showed higher infiltration levels in RBM10 deficient LUADs, while CD4+T cells showed lower infiltration levels in these cases, suggesting that the elevated immune activity in RBM10 deficient LUADs specifically targeted cancer cells.

**Figure 5:**
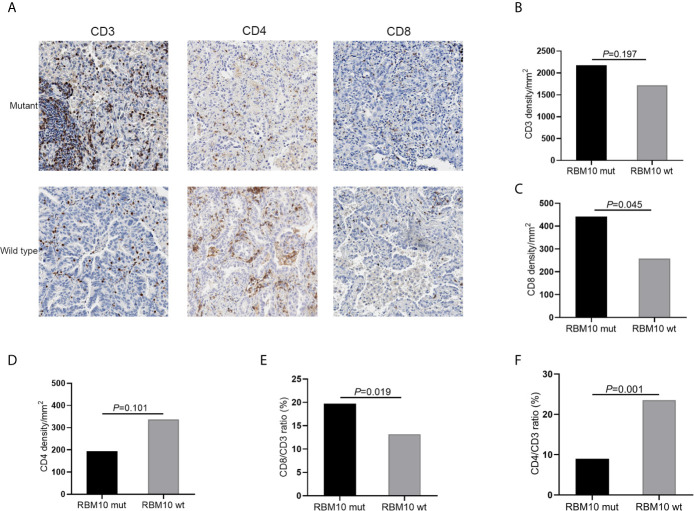
IHC analyses of T cell infiltration in FFPE tumor samples of GGN cohort. **(A)** Representative images of CD3, CD4 and CD8 immunostaining. **(B–D)** CD3+T cell density **(B)** and CD8+T cell density **(C)** are higher in the RBM10 mutant LUADs, and CD4+T cell density is lower in the RBM10 mutant group **(D)**. **(E, F)** CD8/CD3 ratio is higher in RBM10 mutant group than in the wild-type group **(E)**, and CD4/CD3 ratio is lower **(F)**.

To further confirm these results, we performed IHC analyses of CD3+, CD4+ and CD8+ T cells in FFPE samples from the GGN cohort. Representative images of CD3, CD4 and CD8 immunostaining of RBM10 mutant and wild-type LUADs were shown in **Figure 5A**. The CD3+ T cell density was comparable in the RBM10 mutant and wild-type groups (*P*=0.197, **Figure 5B**), suggesting that the level of overall T cells is similar in both groups. However, the CD8+ T cell density was significantly higher (*P*=0.045, **Figure 5C**), indicating that RBM10 mutated patients recruited more T cells for tumor surveillance, while CD4+ T cell density presented no difference between RBM10 mutant and wild-type group (*P*=0.101, **Figure 5D**). In addition, CD8/CD3 ratio was higher in RBM10 mutant group (*P*=0.019, **Figure 5E**), and CD4/CD3 ratio was lower (*P*=0.001, **Figure 5F**), which further proved that the fraction of anti-tumor T cells are higher in patients with RBM10 mutations.

### RBM10 Deficiency Is Associated With Many Immune Markers

**Figure 6:**
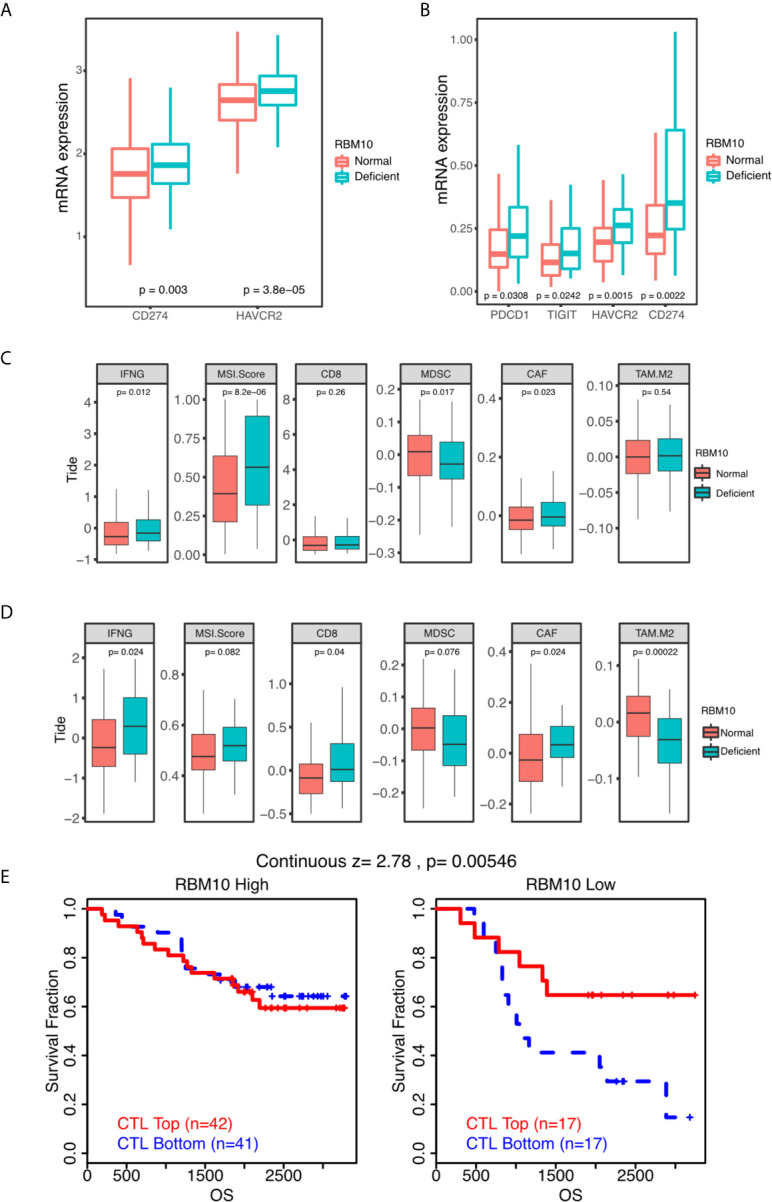
RBM10 deficiency is associated with many immune markers. **(A, B)** Quantitative analysis of mRNA expression of immune checkpoint molecules of between RBM10 deficient LUADs and those with normal expression in TCGA_LUAD **(A)** and CHOICE_ADC **(B)** cohorts. **(C, D)** Several predicted TIDE scores are dysregulated in RBM10 deficient LUADs in both TCGA_LUAD **(C)** and CHOICE_ADC **(D)** cohorts. **(E)** The impacts of RBM10 expression on the effect of cytotoxic T lymphocytes based on survival data of a Japanese LAUD cohort from online website of TIDE.

As LUADs with RBM10 deficiency exhibited higher expression of HLA molecules and higher immune infiltration levels, we speculated if they were also associated with immune markers, especially well-known immune checkpoints. To verify our hypothesis, we compared expression of immune checkpoint molecules between LUADs with RBM10 deficiency and those with normal expression. In the TCGA_LUAD cohort, PD-L1 (CD274, *P*=0.003) and TIM-3 (HAVCR2, *P*<0.001) were highly expressed in RBM10 deficient population (**Figure 6A**). Similarly, PD1 (PDCD1), PD-L1, TIM-3 and TIGIT were all highly expressed in LUADs with RBM10 deficiency in the CHOICE_ADC cohort (all *P*<0.05, **Figure 6B**). Additionally, the predicted score of immunotherapy markers was calculated through TIDE algorithm. IFNG expression and MSI score were higher in RBM10 deficient group in both TCGA_LUAD (**Figure 6C**) and CHOICE_ADC (**Figure 6D**) cohorts. On the contrary, MDSC and M2 macrophage, which played immunosuppressive roles, were lower in RBM10 deficient group (**Figures 6C, D**). These results indicated that RBM10 deficiency is associated with multiple immunotherapy markers.

Furthermore, we obtained survival data of a Japanese cohort of 117 LUADs from online website of TIDE to test whether RBM10 expression had impacts on the effect of cytotoxic T lymphocytes. As shown in **Figure 6E**, patients with high infiltration of cytotoxic T lymphocytes showed better prognosis in RBM10 deficient group. But in RBM10 high expression group, the cytotoxic T lymphocytes level has no impact on survival. These results show that only in RBM10 deficient patients, the anti-tumor function of CTL would present better outcomes.

## Discussion

Recent large-scale genomic sequencing studies have demonstrated that RBM10 is one of the most frequently mutated genes in LUAD and its mutations generally result in loss-of-function (11, 29). Several studies have indicated that RBM10 could suppress lung cancer progression by controlling alternative splicing of other genes. Here, we revealed that RBM10 deficiency had a significant positive association with anti-tumor immunity in LUAD, which implied a novel role of RBM10 in the progression of LUAD.

Notably, RBM10 exhibits a higher mutated frequency in our GGN cohort than in the TCGA_LUAD and CHOICE_ADC cohorts, both of which contained a large fraction of patients with locally advanced or late-stage LUAD. This observation was consistent with the study by Chen et al. which uncovered that RBM10 was significantly more frequently mutated in pre/minimally invasive LUADs than in invasive lesions (10). Another recent study also reported that RBM10 was mutated in 16% of 154 early-stage LUADs, which presented as subsolid nodules on CT scans (13). Therefore, we concluded that RBM10 is more likely to be mutated at the early stage of LUAD.

Cancers accumulate mutations throughout their development and mutations can drive the evolution of cancer. However, certain mutations can also hinder tumor evolution if they trigger anti-tumor immune response, *via* generating neoantigens which are presented on the surface of tumor cells and recognized as “nonself” by adaptive immune system (30, 31). Thus, the immune system is one of the major determinants of tumor evolution. Immune cells can kill the antigenic cells, which is named as immune elimination (32). During tumor evolution, immune elimination represents a negative selective pressure. In this study, we found that the number of neoantigens per mutation was slightly higher in tumors with RBM10 mutations, and clones with RBM10 mutations harbor more neoantigens, but they all didn’t reach statistical significance (*P*=0.15 and 0.12, respectively, **Figures S2D, S2E**), possibly due to the small sample size. Additionally, more neoantigens were generated in clones containing RBM10 mutations in the 11 tumors with RBM10, but the p value only reached marginal statistical significance (*P*=0.064, **Figure S2F**). Our analysis indicated that more neoantigens might be generated in clones containing RBM10 mutations in samples from LUADs with RBM10 mutations. Additionally, RBM10 deficient population exhibited enhanced anti-tumor immunity. We hypothesize that the loss of tumor suppressor function of RBM10 could be crucial for tumorigenesis at the early stage of LUAD, but during the progression of LUAD, RBM10 deficiency activates anti-tumor immunity. Then, tumor clones containing RBM10 mutations may be swept by immune system, resulting in the replacement of RBM10 by other genes. This might explain why the mutation rate of RBM10 was lower in the advanced stage of LUAD than in the early stage. However, it is only a hypothesis, and further studies are needed for validation.

In recent years, immunotherapy has significantly altered the landscape of cancer treatment, including LUAD. Awad et al. indicated that first-line pembrolizumab, one of immune checkpoint blockades (ICB) targeting PD-1, plus pemetrexed-carboplatin continued to show improved response and survival versus chemotherapy alone in advanced non-squamous non-small cell lung cancer (NSCLC) (33). Durvalumab, a selective human IgG1 monoclonal antibody blocking PD-L1, could also prolong overall survival of Stage III NSCLC after chemoradiotherapy (34). However, only a minority of unselected patients could really benefit from immunotherapy. Therefore, it is particularly important to identify efficient biomarkers for screening the dominant population of immunotherapy. Previous studies have indicated that PD-L1 expression and TMB can serve as predictive markers for the efficacy of ICBs. However, certain limitations exist. For example, PD-L1 expression exhibits significant spatial and temporal heterogeneity and the utilization of TMB is limited due to the lack of a uniform standard to determine the cut-off value (35, 36). Additionally, even with high PD-L1 expression or TMB, some patients still don’t show response to ICBs. Abundant studies indicate that the efficiency of cancer immunotherapy is associated with certain genetic features, including mutations of specific genes. Zhang et al. found that ZFHX3 mutations were closely related to longer overall survival in NSCLC patients treated with ICBs and suggested that ZFHX3 mutations could be used as a novel predictive marker in guiding NSCLC ICB treatment (37). Recently, EPHA mutations were verified to be an independent classifier that could stratify patients with LUAD for ICB treatment (38). In this study, we demonstrated that RBM10 deficient population had higher TMB, and exhibited higher HLA and immune infiltration levels. These indicated RBM10 deficiency could enhance immunogenicity of LUADs. Therefore, we think that LUADs with RBM10 deficiency may be a potential population with well response to immunotherapy, but more researches are still needed.

There were several limitations to our study. First, due to the small sample size of the GGN cohort and the lack of RNA-seq data, we could only employ data from TCGA and publicly available datasets to perform several bioinformatic analyses in this study. Second, although RBM10 deficiency could enhance the immunogenicity of LUADs, indicating these patients could potentially benefit from immunotherapy, future studies that would examine this hypothesis directly are needed to confirm the findings of our bioinformatic analyses.

## Conclusion

This study found that RBM10 deficient LUADs had higher TMB, exhibited higher HLA and immune infiltration levels. In brief, RBM10 deficiency could enhance anti-tumor immunity in LUAD.

## Data Availability Statement

The datasets presented in this study can be found in online repositories. The names of the repository/repositories and accession number(s) can be found below: http://bigd.big.ac.cn/gsa-human, HRA000044 and HRA000970.

## Ethics Statement

The studies involving human participants were reviewed and approved by Ethics Committee of Peking University Cancer Hospital & Institute (Institutional Review Board No. 2019KT59). The patients/participants provided their written informed consent to participate in this study.

## Author Contributions

Study design: BL, HW, YW, XX, JL, and NW. Bioinformatic analysis: HW, XG, YG, and XYi. Experiments: ZL, LY, XYa, and YM. Manuscript writing: BL and HW. Manuscript revision: SY, JL, and NW. Supervision: XX and NW. Funding: NW. All authors contributed to the article and approved the submitted version.

## Funding

This study was supported by National Key R&D Program of China (No. 2018YFC0910700), National Natural Science Foundation of China (No. 81972842), Beijing Natural Science Foundation (No. 7192036), Special Fund of Beijing Municipal Administration of Hospitals Clinical Medicine Development (No. XMLX201841), Beijing Municipal Administration of Hospital’s Ascent Plan (No. DFL20191101).

## Conflict of Interest

Authors HW, XG, YG, XY and XX are employed by Geneplus-Beijing.

The remaining authors declare that the research was conducted in the absence of any commercial or financial relationships that could be construed as a potential conflict of interest.
