# Supplementary figures and images for: RBM10 Deficiency Is Associated With Increased Immune Activity in Lung Adenocarcinoma
Source: Front Oncol. 2021 Jul 21;11:677826. doi: 10.3389/fonc.2021.677826 (PMC8336464; doi:10.3389/fonc.2021.677826)

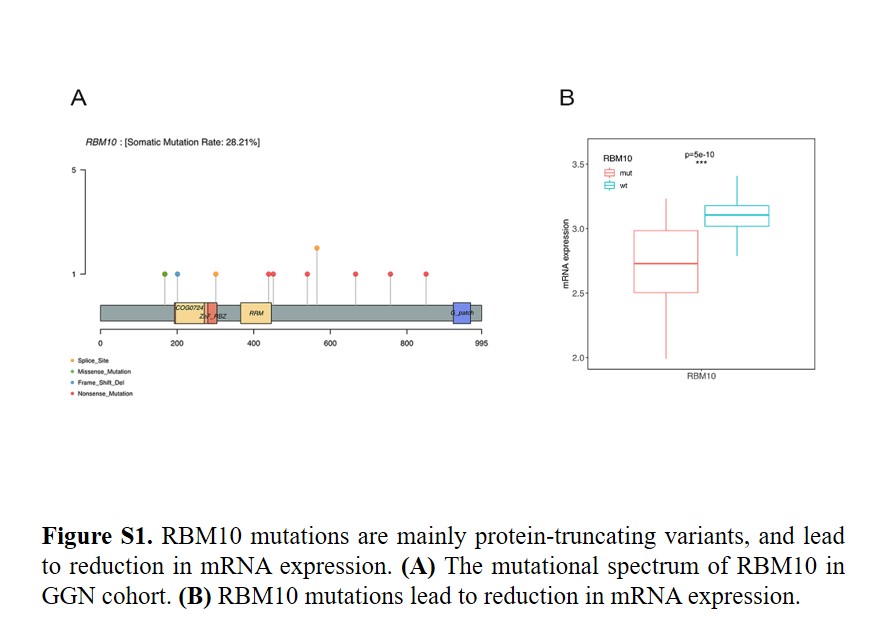

Supplement: Supplementary file 1 [file Image_1.jpg]

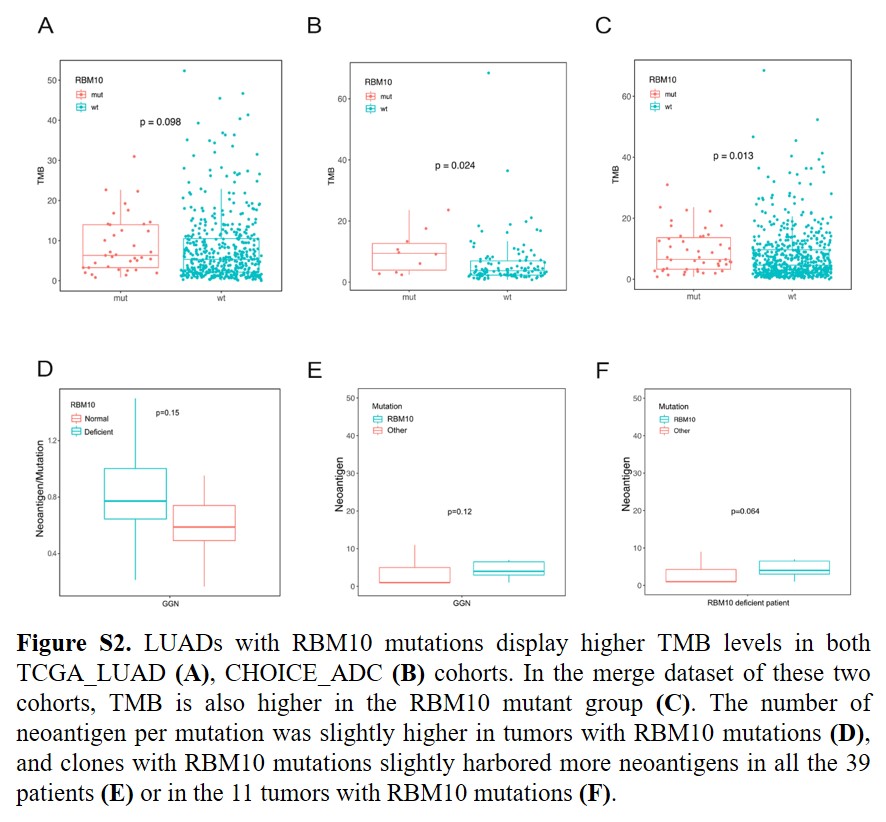

Supplement: Supplementary file 2 [file Image_2.jpg]
